# Mechanisms of Memory Updating: State Dependency vs. Reconsolidation

**DOI:** 10.5334/joc.198

**Published:** 2022-01-07

**Authors:** Christopher Kiley, Colleen M. Parks

**Affiliations:** 1 University of Nevada, Las Vegas, US

**Keywords:** reconsolidation, state dependency, episodic memory, memory updating, integration concept

## Abstract

Reactivating a memory trace has been argued to put it in a fragile state where it must undergo a stabilization process known as reconsolidation. During this process, memories are thought to be susceptible to interference and can be updated with new information. In the spatial context paradigm, memory updating has been shown to occur when new information is presented in the same spatial context as old information, an effect attributed to a reconsolidation process. However, the integration concept holds that memory change can only occur when reactivation and test states are the same, similar to a state-dependent effect. Thus, in human episodic memory, memory updating should only be found when state is the same across the study, reactivation, and test sessions. We investigated whether memory updating can be attributed to state dependency in two experiments using mood as a state. We found evidence of memory updating only when mood was the same across all sessions of the experiments, lending support to the integration concept and posing a challenge to a reconsolidation explanation.

Reconsolidation theory holds that activation of an existing memory trace can destabilize it, which opens a time-dependent window during which the memory is fragile. The memory can then be strengthened, weakened, or even altered, before it is restabilized, or reconsolidated (see Elsey et al., 2018 for a review). Much of the evidence for reconsolidation comes from nonhuman animal studies using a three-session fear memory paradigm. In these three-session studies, rodents develop a fear memory in session 1, have that memory reactivated in session 2 by re-exposure to the fear context (without the fear-inducing stimulus), followed by the administration of a protein synthesis inhibitor or a control vehicle, and are then tested on the fear memory in session 3. Evidence of reconsolidation has typically come in the form of retrograde amnesia in the protein synthesis inhibition condition and intact memory in the control condition; that is, the original fear memory is weakened (possibly even erased) by blocking the protein synthesis thought to be necessary to reconsolidate the memory, but is intact in the control condition (e.g., Nader et al., 2000; see Haubrich et al., 2020 for a review). Thus, the fear memory can be weakened to the point that it is no longer recoverable due to a reconsolidation blockade (but see Trent et al., 2015), providing evidence of a process that is necessary to restabilize memories after reactivation.

Evidence of reconsolidation in human declarative memory looks quite different. In one of the first studies to examine reconsolidation in human episodic memory, Hupbach et al. (2007) asked participants to memorize a set of objects in one session. Two days later, participants were either reminded of the Day-1 procedure by returning to the same space and explaining the Day-1 procedure, or were not reminded (they went to a different space and were not asked about Day 1). They then learned a new set of items. On Day 3 (another 48 hours later), both groups were given a free recall test of Day 1 items. Participants who received a reminder of Day 1 before learning the second list made significantly more intrusions from that list on the recall test than the no-reminder group. Furthermore, when tested on List 2 instead of List 1, there was no difference in intrusions between the reminder and no-reminder conditions in List 1 intrusions, resulting in an asymmetrical pattern of intrusions across experiments; namely, List 2 intruded on List 1, but there was no evidence of List 1 intruding on List 2. Thus, it was thought that the reminder (primarily spatial context) was critical to triggering the updating process, and this pattern was taken as evidence of reconsolidation of the first list. Several studies have replicated these results using this three-day spatial context paradigm (Scully et al., 2017; but see Klingmüller et al., 2017). Reactivating an established memory by having subjects study objects in the same (previously unfamiliar) space across Days 1 and 2, versus different spaces, leads to memory updating (Hupbach et al., 2008), although it does not have an effect on the accuracy of recall of List 1. So rather than producing a weakened memory as is found in the nonhuman animal literature, reactivation + interference produces an altered memory, one that has been updated with new information.

## Reconsolidation vs. Memory Integration

Reconsolidation theory holds that memory change is due to new protein synthesis (or lack thereof) during a period of time between reactivation and the final test of the original memory. Blocking this synthesis, as is done in nonhuman animals, has been found to result in memory loss; it is assumed, then, that the inhibitor has blocked the reconsolidation process necessary to restablize a reactivated (and fragile) memory. In human declarative memory, it is thought that presenting interfering information shortly after reactivation is what produces memory change, typically updating. The literature on human episodic memory appears to tacitly assume that the reconsolidation process found in nonhuman animals is similar, if not the same as, that found in humans, or more specifically that reconsolidation is a process of new protein synthesis in all animals. Because this synthesis takes time, the results of the reactivation + treatment manipulation should only be found after a delay between reactivation and the final test; that is, neither memory loss nor memory updating should be found immediately after the reactivation + treatment manipulation. This time dependency has been found in both rodent fear memory and in human declarative memory (e.g., Hupbach et al., 2007), lending support to the reconsolidation theory (but see Gisquet-Verrier & Riccio, 2012 for evidence to the contrary).

Nonetheless, reconsolidation theory’s explanation of memory change faces some challenges. The memory integration concept (Gisquet-Verrier & Riccio, 2018, 2019a) argues that effects such as updating or weakening are due to memory integration and state dependency. Specifically, rather than a memory trace starting out as fragile (or becoming fragile again upon reactivation), traces are said to be malleable during a particular window of time starting shortly before the memory forms or is reactivated and ending shortly afterward. Traces are rapidly updated, which can lead to the incorporation of new content (i.e., updating), strengthening (e.g., with an additional learning trial), weakening (e.g., due to the administration of an amnestic agent), or dramatic changes (e.g., false memory). Although both views agree that established memories can be changed when reactivated, memory integration differs from reconsolidation theory in that: (1) a new or reactivated memory trace is not fragile in the sense that it needs to be restabilized in order to avoid its complete loss, but is malleable in the sense that it can be changed; (2) the time window of malleability starts shortly before the memory is reactivated rather than shortly after; (3) creation of new memories and updating of reactivated memories does not require new protein synthesis; and (4) new information is rapidly integrated with the information retrieved from the original trace, and thus updating effects are not reliant on a delay period between reactivation + treatment and the final test (e.g., Gisquet-Verrier & Riccio, 2012; see Bridge & Voss, 2014, Bridge & Paller, 2012 for evidence in human declarative memory). Thus, the integration concept ultimately argues that there is no reconsolidation process at all.

If reconsolidation is not real, to what can we attribute effects that appear to be due to disrupting reconsolidation? The memory integration concept argues that effects thought to be due to reconsolidation are in fact due to state dependency, much like Tulving and Thompson’s (1973) encoding specificity effect or context dependent effects (e.g., Godden & Baddeley, 1975; see Smith & Vela, 2001 for a review). Specifically, materials learned in a particular state or context are better remembered when in that same state or context at test than in a different one. For instance, working with rats, Gisquet-Verrier et al. (2015) found that the injection of a protein synthesis inhibitor during the reminder period acted as a state manipulation. When animals were in *different* states between the reminder period and final retrieval, they found impaired memory; the state change made it difficult to retrieve the memory at the final test, thus appearing as though the memory was weakened or lost altogether. However, when they were in the *same* state during the reminder and test (i.e., they were injected with the protein synthesis inhibitor during reactivation *and* at test), there was no evidence of memory change; that is, memory was found to be intact. According to the integration concept, there was no state change between reactivation and test in that condition, and thus there were no retrieval difficulties. Because the majority of reconsolidation studies in nonhuman animals have used protein synthesis inhibitors at reactivation but not at test, Gisquet-Verrier et al. argued that the results found in past experiments are actually state-dependent effects.

**Figure 1:**
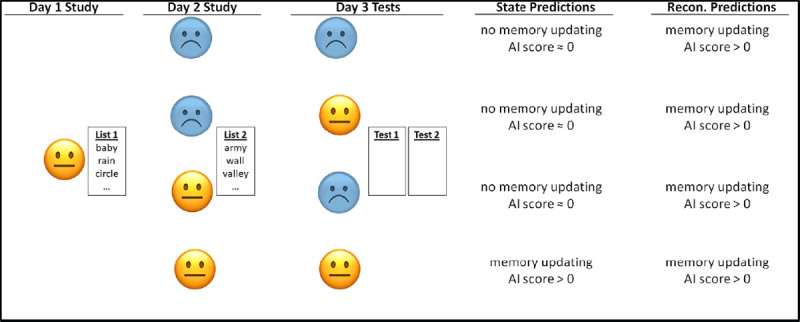
Mood manipulation design of Experiment 1. No mood was induced on Day 1; it was assumed to be neutral.

The aim of the current research was to test this idea in human episodic memory using the spatial context paradigm (Hupbach et al., 2007) to look at memory updating. Evidence from this paradigm suggests that there is something special about space as a reminder; whereas space has been consistently effective in reactivating Day-1 memory and triggering reconsolidation, other reminders have not been effective (Hubach et al., 2008; Kiley & Parks, 2020). Thus, according to research with this paradigm, one should see updating when space is held constant across the three days. According to a state-dependency account, however, memory change should be seen only when the person’s state is the same on all three days (see ***Figure 1***).

To test these predictions, we measured asymmetrical intrusions (the AI score) between tests of List 1 and of List 2. The AI score speaks directly to the asymmetry of intrusions, which is key for reconsolidation theory in the spatial context paradigm (Kiley & Parks, 2020). The intrusions measured in the AI score are List-2 responses on the test of List 1, and List-1 responses on the test of List 2. The AI score is the difference between the number of intrusions from List 2 into List 1 (the score typically reported) and the number of intrusions from List 1 into List 2 ((List 2 → List 1) – (List 1 → List 2)). For instance, if a participant intruded three items from List 2 into List 1 and one item from List 1 into List 2, they would have an AI score of two, indicating that memory for List 1 was updated with items from List 2 more so than in the other direction. Positive AI scores that are significantly different from zero reflect memory updating, values near zero reflect no updating, and significantly negative AI scores would reflect more List 1 intrusions into List 2 than vice versa (likely a source misattribution effect). This measure improves upon previous dependent variables because it includes intrusions on both tests and thus directly speaks to asymmetry. Previous studies that have investigated the directionality of intrusions have typically found intrusions from List 2 into List 1 and not in the other direction, but have not tested both lists in a single experiment (e.g., Hubach et al., 2007), which is needed to argue for asymmetrical updating. If reconsolidation theory explains updating, we would expect to see AI scores greater than zero in all conditions (and there would be no reason to think that they would differ across conditions). If updating is attributable to state dependency though, we should see that AI scores would be greater than zero only when mood state is the same across all three days (see ***Figure 1***).

## Experiment 1

In Experiment 1, we tested the state dependency hypothesis by manipulating participants’ moods in the three-day spatial-context paradigm. Participants were always tested in the same room by the same experimenter. If the reconsolidation account of updating in the spatial-context paradigm is correct, participants in all conditions would show memory updating due to the fact that spatial context remained the same on all three days (i.e., there was a spatial reminder in all conditions). However, if the updating effect is due to state dependency, as predicted by the integration concept, it would appear only when mood states match on all three days (see ***Figure 1*** for procedure and predictions).

## Method

### Participants

Power analyses using G*Power3.0 were conducted with the effect size reported by Scully, et al. (2017) for a comparison between reminder and no-reminder conditions, Cohen’s d = 1.03. The power analysis indicated that 13 participants were needed per condition. We tested slightly more participants because 13 was not sufficient for our counterbalance, and the power analysis used Cohen’s d for t-tests rather than η_p_^2^ for the F tests that we used. Participants were excluded from the data analyses if they did not follow instructions (e.g., recalled substantially more extra-list intrusions than list items) or if they copied more than five items from the first test onto the second test on Day 3 (see procedure). Three participants were excluded for having more than five copies, but all participants followed the instructions. Data were analyzed for 64 participants (19 male, 45 female, age M = 19.60, SD = 2.10) recruited from the University of Nevada, Las Vegas. They were randomly assigned to one of four conditions for a total of 16 participants per condition.

### Materials

We used 50 unrelated neutral words to create two lists of 25 words each. Words were of middle to high frequency (List 1 M = 103; List 2 M = 102) and between four and five letters each (List 1 M = 4.76; List 2 M = 4.88). Lists were counterbalanced such that each list appeared equally often as the learning material on Days 1 and 2.

Negative mood was induced using the Baker and Guttfreund (1993) procedure, in which participants are asked to conjure negative emotions by calling to mind the most negative event in their life and writing about it for 10 minutes. For neutral mood induction conditions, participants read articles about the geography of various land masses taken from Wikipedia and answered a question about how they felt while reading them. The Positive and Negative Affect Schedule (PANAS, Watson et al., 1988) was administered after the mood induction as a manipulation check.

### Procedure

Participants came to the lab for three sessions that took place 24 hours apart and were tested in the same space with the same experimenter on all three days. On Day 1, participants were shown words one at a time on a computer screen for 2000 ms each, followed by a free recall test over the words. Following Hupbach et al. (2007), participants had to recall at least 80% of the words to ensure that they were sufficiently encoded. If they recalled less than 80% of the words correctly, the list was presented again, followed by another free recall task. This cycle repeated up to five times. If a participant failed to reach criterion after the fifth attempt, the experiment concluded. Mood was not manipulated on Day 1.

On Day 2, half of the participants took part in the Autobiographical Recollection Induction Procedure (Baker & Guttfreund, 1993) to induce a negative mood. Participants in this condition spent 10 minutes reflecting on one of the saddest events in their life. The rest of the participants read a two-page article on a geographical region of the US, were asked to explain in a few words how the passage made them feel, and were then administered the PANAS. After the PANAS manipulation check, both groups learned the second list of words.

On Day 3, we further split participants into two conditions, creating a 2 × 2 design for the experiment (see ***Figure 1***). Half the participants were given the negative mood induction (remembering a different sad event in their life if they had gone through the induction on Day 2) and half read a geography article; this was followed by the PANAS. Participants were then given source-specific free recall tests over the words presented on Days 1 and 2. Day-1 words were always tested first, to get a more conservative estimate of the reconsolidation effect, as we were most interested in the intrusions made from Day 2 into Day 1.

## Results and Discussion

### Statistical Analyses

We used analysis of variance (ANOVA), planned comparisons (t-tests, with alpha adjusted for the number of comparisons), and post hoc comparisons (t-test with adjusted alpha values) to analyze the results using null hypothesis testing. We rely on effect sizes to interpret the results as well, and use Cohen’s d for the t-test effect size and η_p_^2^ (partial eta squared) for the ANOVA effect size. Statistics were computed with SPSS 26 with alpha set at .05 unless noted otherwise.

### Manipulation Check

A difference score between positive and negative affect ratings on the PANAS was compared between those in the negative and neutral conditions for Days 2 and 3 with one-tailed t-tests (see ***Table 1***). The difference scores were significantly lower in the negative than in the neutral condition for Day 2, *t*_(62)_ = –3.81, *p* < .001, Cohen’s d = .953, and Day 3, *t*_(62)_ = –3.45, *p* < .001, d = .863, indicating a significant difference in mood, with more negative affect in the negative condition.

**Table 1:** Mean PANAS difference scores (and standard deviations).


	EXPERIMENT 1			EXPERIMENT 2		
	
	DAY 2	DAY 3		DAY 1	DAY 2	DAY 3

Negative	.17	.26		.20	.06	.35

	(1.25)	(1.34)		(1.07)	(1.22)	(1.30)

Neutral	1.28	1.30			1.22	.99

	(1.08)	(1.04)			(.89)	(.66)


### Acquisition Performance Days 1 and 2

Due to a computer crash, data files for Day-1 and Day-2 acquisition were missing for two participants. Participants reached at least 80% accuracy on Days 1 and 2 (see ***Table 2*** for means). A 2 × 2 × 2 mixed ANOVA (Day-2 mood × Day-3 mood × Day) was conducted to compare the number of blocks needed to reach criterion for Day 1 and Day 2. There was a main effect of Day, *F*_(1, 58)_ = 8.67, *p* = .005, η_p_^2^ = .130, and a significant interaction between Day 2 and Day 3 mood, *F*_(1, 58)_ = 6.49, *p* = .014, η_p_^2^ = .101. The main effect of Day indicates that participants learned the lists with fewer attempts on Day 2, likely an effect of practice with the task. The interaction showed that those in a neutral mood on both days took longer to reach criterion than those in the other combinations of mood conditions. No other effects were significant, *F*s < 2.5, *p*s > .13.

**Table 2:** Means (and standard deviations) of blocks to acquisition on Days 1 and 2 in Experiment 1.


	BLOCKS DAY 1			BLOCKS DAY 2	
	
	DAY 2 MOOD			DAY 2 MOOD	
	
	NEGATIVE	NEUTRAL		NEGATIVE	NEUTRAL

Day 3 Mood					

Negative	2.80	2.60		2.40	2.50

	(.68)	(.99)		(.99)	(.89)

Neutral	2.38	3.13		2.13	2.81

	(1.09)	(.50)		(.72)	(.66)


### Day 3 Free Recall Accuracy

A 2 (Day-2 mood) × 2 (Day-3 mood) × 2 (List) mixed ANOVA, with List as the repeated measure, revealed a main effect of List, *F_(1, 60)_* = 13.21, *p* = .001, η_p_^2^ = .180; no other effects were significant, *F*s < 3.5, *p*s > .07. Expectedly, recall was better for List 2 than for List 1, List 1 M = 10.73 (SD = 4.94), List 2 M = 13.33 (SD = 4.88). Though recall of List 1 was lower than that of List 2 (see ***Table 3***), there was no reliable evidence that the mood manipulation affected the accuracy of recall of either list (i.e., there was no state effect on accurate recall).

**Table 3:** Mean free recall accuracy (and standard deviations) on Day 3 for Experiment 1.


	ACCURACY LIST 1			ACCURACY LIST 2	
	
	DAY 2 MOOD			DAY 2 MOOD	
	
	NEGATIVE	NEUTRAL		NEGATIVE	NEUTRAL

Day 3 Mood					

Negative	11.25	9.13		12.50	13.81

	(5.14)	(3.54)		(5.11)	(4.61)

Neutral	12.25	10.31		15.31	11.69

	(5.73)	(5.17)		(5.42)	(3.91)


### Asymmetrical Intrusion Score

The main variable of interest, the asymmetrical intrusion (AI) score, was calculated by creating a difference score between the number of intrusions from List 2 → 1 and List 1 → 2. In many reconsolidation studies, List 2 is not tested. Thus, it is not clear if the intrusions are always asymmetric or if there are just more intrusions found in the condition in which List-1 memory is reactivated (i.e., the reminder condition). If the observed pattern of intrusions is truly asymmetrical, then a positive AI score is produced. An AI score that does not differ from zero would demonstrate an equivalent number of intrusions in both directions, and a negative score would demonstrate more List-1 intrusions into List-2 recall than vice versa.

**Figure 2:**
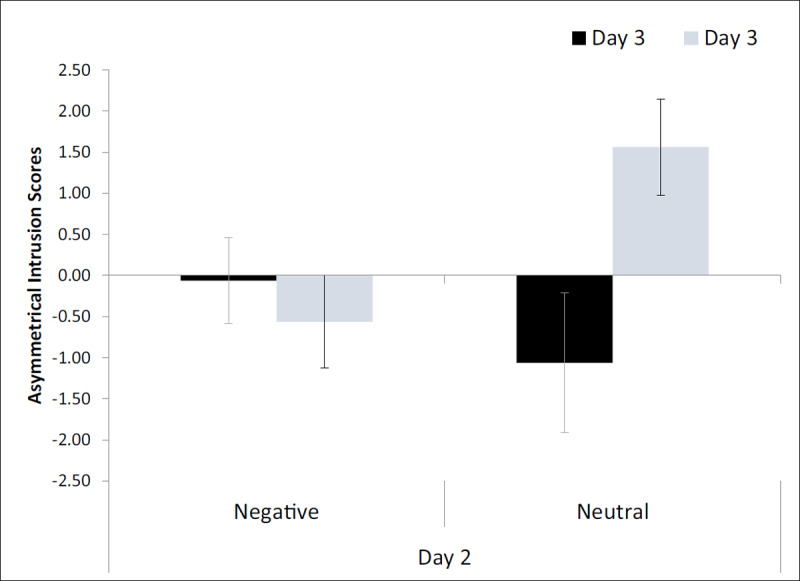
AI scores as a function of mood on Day 2 and Day 3 in Experiment 1. Error bars represent one standard error.

The AI scores (***Figure 2***, see ***Table 4*** for raw intrusions in both lists), were analyzed using a two-way ANOVA (Day-2 mood × Day-3 mood). Although the main effects were not significant, *F*s < 2.7, *p*s > .100, the interaction was *F_(1, 60)_* = 5.83, *p* = .019, η_p_^2^ = .089. Planned one-sample t-tests (α = .0125) indicated that the AI score was only significantly different from zero when participants were in a neutral mood state on both Day 2 and Day 3 *t_(15)_* = 2.67, *p* = .009, Cohen’s d = .669 (other –.12 < *t*s < 1.25, *p*s > .100). We also conducted post hoc tests (α = .025) to determine whether the differences between the Day 3 conditions in each Day 2 condition were reliable. T-tests comparing Day-3 negative and neutral states, in the Day-2 *negative* condition (adjacent bars on the left in ***Figure 2***), produced no significant difference, *t*_(30)_ = .64, *p* = .528. Comparison between Day-3 negative and neutral states, in the Day 2 *neutral* condition (adjacent bars on the right in ***Figure 2***), produced a significant difference, with a higher AI score for Day 3 neutral than Day 3 negative, *t*_(30)_ = –2.55, *p* = .016.

**Table 4:** Mean raw intrusions (and standard deviations) for List 2 into List 1 and List 1 into List 2 as a function of mood sate. Mood was (assumed) neutral on Day 1 in all conditions.


	LIST 2 → LIST 1 INTRUSIONS			LIST 1 → LIST 2 INTRUSIONS	
	
	DAY 2 MOOD			DAY 2 MOOD	
	
	NEGATIVE	NEUTRAL		NEGATIVE	NEUTRAL

Day 3 Mood					

Negative	2.69	2.38		2.75	3.44

	(2.89)	(2.25)		(3.73)	(2.56)

Neutral	1.75	3.94		2.31	2.38

	(2.59)	(2.54)		(1.85)	(2.19)


It can also be argued that the groups that speak best to the question at hand are the neutral-neutral group and the negative-neutral group because the other groups included test conditions that differed in mood from Day 1 (which we assume was neutral). When acquisition and test moods were held constant, the size of the effect appears even stronger than what was indicated in the interaction. This can be seen in the AI scores (the light grey bars in ***Figure 2***) as well as in the raw intrusion rates, with neutral-neutral at M = 3.94 and negative-neutral at M = 1.75 (***Table 4***). Indeed, post hoc comparisons (one-tailed, α = .025) revealed a significant difference between these two conditions for the AI scores *t*_(30)_ = –2.62, *p* = .007, Cohen’s d = .926, as well as the raw intrusions, *t*_(30)_ = –2.14, *p* = .011, Cohen’s d = .852, both large effect sizes. Thus, regardless of how it is compared, it is clear that when mood is the same across days, AI scores and intrusion rates are positive and greater than when mood differed on Day 2.

These data show a memory-updating effect only in the condition where mood was neutral on both Day 2 and Day 3. Crucially, it is also the only condition where mood was kept constant across all three days of the experiment, since mood was likely neutral on Day 1 in all conditions. However, because no mood was induced on Day 1, we do not know definitively that participants were in a neutral emotional state. Experiment 2 was conducted to address this issue.

## Experiment 2

Experiment 2 was designed to test the hypothesis that the reconsolidation pattern is only found when mood state is the same on all three days of the spatial context paradigm. Rather than having all participants learn the first list in a presumably neutral mood, all participants learned Day-1 words after undergoing the negative mood induction.

## Method

### Participants

Data from three subjects were removed from the analyses due to copying more than five words from test 1 onto test 2, and another two were excluded for failing to follow instructions (they recalled substantially more extra-list intrusions than list items, e.g., 22 vs. 4). Data from 64 participants (23 male, 40 female, 1 nonbinary; age M = 19.47, SD = 2.60) recruited from the University of Nevada, Las Vegas were analyzed. Subjects were randomly assigned to one of four conditions for a total of 16 participants per condition.

### Materials and Procedure

The procedure and materials were the same as those used in Experiment 1, with the exception that all participants went through the negative mood induction on Day 1 prior to learning.

## Results and Discussion

### Manipulation Check

The PANAS difference scores were significantly lower in the negative than in the neutral condition for Day 2, *t*_(62)_ = –4.33, *p* < .001, Cohen’s d = 1.082, and Day 3, *t*_(62)_ = –2.49, *p* < .001, d = .623, indicating a difference in mood in the two conditions on both days (see ***Table 1***).

### Acquisition Performance Days 1 and 2

Due to the same computer crash noted previously, data files for Day-1 and Day-2 acquisition were missing for six participants. Participants reached at least 80% accuracy on Days 1 and 2 (see ***Table 5*** for means). A 2 × 2 × 2 mixed ANOVA (Day-2 mood × Day-3 mood × Day) was conducted to compare the number of blocks needed to reach criterion for Day 1 and Day 2. There was a main effect of Day, *F*_(1, 54)_ = 7.26, *p* = .009, η_p_^2^ = .119, indicating that participants learned the lists with fewer attempts on Day 2, likely an effect of practice with the task. No other effects were significant, *F*s < 1.30, *p*s > .27.

**Table 5:** Means (and standard deviations) of blocks to acquisition on Days 1 and 2 in Experiment 1.


	BLOCKS DAY 1			BLOCKS DAY 2	
	
	DAY 2 MOOD			DAY 2 MOOD	
	
	NEGATIVE	NEUTRAL		NEGATIVE	NEUTRAL

Day 3 Mood					

Negative	2.64	2.67		2.29	2.56

	(.63)	(1.05)		(1.27)	(.73)

Neutral	2.94	2.62		2.38	2.36

	(.93)	(.65)		(.62)	(.50)


### Day 3 Free Recall Accuracy

A 2 (Day-2 mood) × 2 (Day-3 mood) × 2 (List) mixed ANOVA, with List as the repeated measure, revealed a main effect of List, *F_(1, 60)_* = 25.48, *p* < .001, η_p_^2^ = .298; no other effects were significant, *F*s < 1.10, *p*s > .30. Expectedly, correct recall (***Table 6***) was better for List 2 than for List 1, but there was no reliable evidence for a difference as a function of mood condition, (i.e., as in the previous experiment, there was no state effect on accurate recall).

**Table 6:** Mean free recall accuracy (and standard deviations) on Day 3 for Experiment 2.


	ACCURACY LIST 1			ACCURACY LIST 2	
	
	DAY 2 MOOD			DAY 2 MOOD	
	
	NEGATIVE	NEUTRAL		NEGATIVE	NEUTRAL

Day 3 Mood					

Negative	9.75	10.75		12.56	14.50

	(6.54)	(3.71)		(5.28)	(4.56)

Neutral	11.25	10.75		13.75	14.94

	(2.82)	(4.23)		(4.04)	(5.08)


### Asymmetrical Intrusion Scores

**Figure 3:**
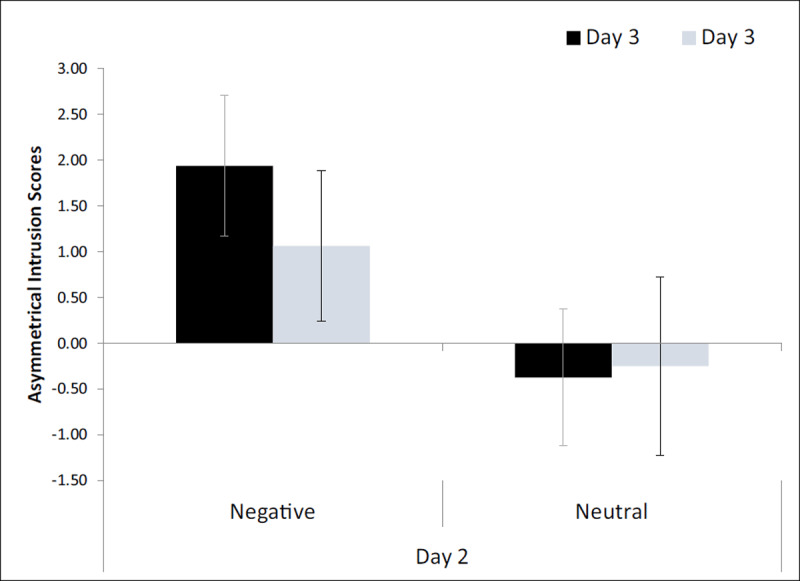
Asymmetrical intrusion scores for Experiment 2. All participants were in a negative mood on Day 1. Error bars represent standard error.

A two-way ANOVA (Day-2 mood × Day-3 mood) revealed a significant effect of Day-2 mood *F_(1, 60)_* = 4.80, *p* = .032, η_p_^2^ = .07, but no other effects were significant (*F*s < 1, *p*s > .50). Thus, participants who were in the negative condition on Day 2 had a higher AI score than those who were in a neutral state on Day 2 (***Figure 3***). This result was primarily driven by participants who were not only in a negative state during Day 2, but during Day 3 as well. One-tailed, single-sample t-tests (α = .0125) showed that the AI score was significantly different from zero only when participants were in a negative mood on all three days, *t*_(15)_ = 2.60, *p* = .010, Cohen’s d = .651 (other –.51 < *t*s < 1.30, *p*s > .100). We also conducted post hoc tests to determine whether the differences between the Day 3 conditions in each Day 2 condition were reliable. A t-test (α = .025) comparing Day-3 negative and neutral states, in the Day-2 *negative* condition (adjacent bars on the left in ***Figure 3***), produced no significant difference, *t*_(30)_ = .10, *p* = .920. Likewise, comparison between Day-3 negative and neutral states, in the Day-2 *neutral* condition (adjacent bars on the right in ***Figure 3***), produced no significant difference, *t*_(30)_ = –.79, *p* = .435. In sum, though not as strong an effect as in Experiment 1, these results show that the asymmetric memory-updating pattern is found when mood was the same on all three days, but there was no significant evidence for such an effect in the other conditions.

Similar to Experiment 1, we compared scores (AI scores and raw intrusions) between the negative-negative-negative and negative-neutral-negative conditions. When acquisition and test moods were the same, the size of the effect appears stronger than what was indicated in the comparisons of the AI scores above. This can be seen in the AI scores (the two black bars in ***Figure 3***) as well as in the raw intrusion rates, with negative-negative-negative at M = 3.00 and negative-negative-neutral at M = 2.13 (***Table 7***). Post hoc comparisons (one-tailed, α = .025) revealed a significant difference between these two conditions for the AI scores *t*_(30)_ = 2.19, *p* = .018, Cohen’s d = .776, although the raw intrusion rates were not significantly different, *t*_(30)_ = .733, *p* = .235. This pattern mimics that found in Experiment 1, showing that when mood is the same across days, AI scores are positive and greater than when mood differed on Day 2.

**Table 7:** Mean raw intrusions (and standard deviations) for List 2 into List 1 and List 1 into List 2 as a function of mood sate. Mood was negative for Day 1 in all conditions.


	LIST 2 → LIST 1 INTRUSIONS			LIST 1 → LIST 2 INTRUSIONS	
	
	DAY 2 MOOD			DAY 2 MOOD	
	
	NEGATIVE	NEUTRAL		NEGATIVE	NEUTRAL

Day 3 Mood					

Negative	3.00	2.13		2.56	2.50

	(4.32)	(2.03)		(2.28)	(1.67)

Neutral	3.19	2.69		2.13	2.94

	(2.48)	(2.30)		(1.75)	(3.09)


## Discussion

**Figure 4:**
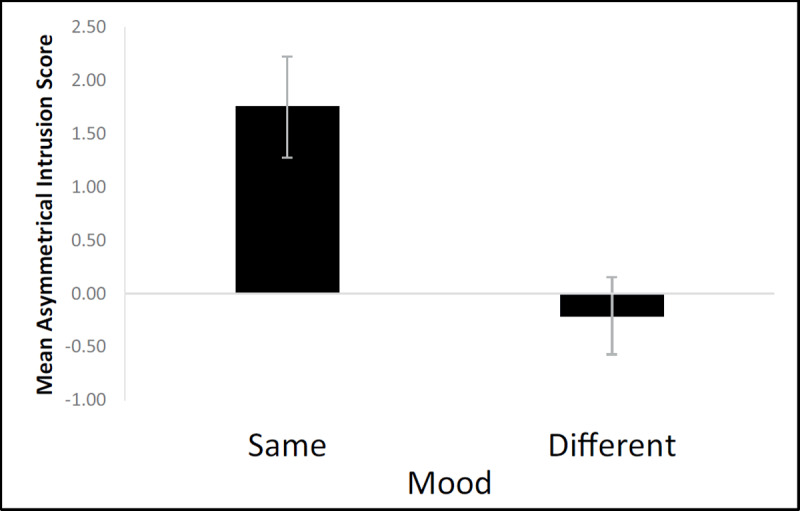
AI scores collapsed across experiments and conditions as a function of mood, which was the same on all three days (n = 32) or differed across days (n = 96), *t_(126)_* = 3.25, *p* < .001, Cohen’s d = .631. Error bars represent one standard error of the mean.

The current study tested a state-dependent explanation of memory updating, namely that memory updating is not due to a reconsolidation process, but is instead an outcome of matches and mismatches of states between study, reactivation, and test. We manipulated mood and found the asymmetrical intrusion pattern only when participants were in the same mood on all three days (see ***Figure 4***, data collapsed across experiments). Although it is difficult to interpret null effects (i.e., the lack of a significant difference from zero in all the different conditions), the differences between the *same* and *different* conditions are quite clear. Given previous findings that spatial context serves as a special reminder, we should have seen memory updating in all of the conditions; that is, the reconsolidation theory predicts that mood should be irrelevant. Instead, our findings support the idea that items are bound to particular states, and that memory updating is most likely to occur when items are bound to the same state, such that the state can serve as part of the retrieval cue compound on both Days 2 and 3 (e.g., Gisquet-Verrier et al., 2015; Klingmüller, et al, 2017; for a similar argument with temporal context, see Sederberg et al., 2011). Thus, these results support the integration concept’s explanation of memory-updating patterns, a more parsimonious explanation than reconsolidation in that it does not require a unique neurobiological process but rests on well-established memory phenomena, specifically, encoding specificity and state effects on memory.

Although several studies have supported the reconsolidation theory (see Elsey et al., 2018 for a review), there are others that have failed to replicate previous findings or failed to support its predictions. For instance, Klingmüller et al. (2017) failed to replicate memory updating effects in the spatial context paradigm, showing that the asymmetrical intrusion pattern appeared only when there was a very drastic change in context between spaces on Day 1 and Day 2 in the no-reminder condition. Interestingly, they also found no evidence to support the idea that the reminder (same spatial context) actually reactivated the original memory at all. Levy et al. (2018) found no evidence of reconsolidation when using a within-subjects design, and Hardwicke et al. (2016) also failed to find an asymmetrical intrusion pattern in declarative memory when using letter and number strings, instead finding that reactivating Day-1 memory produced better memory compared to when it was not reactivated. Our study contributes to this collection, supporting Klingmüller et al. in showing that spatial context does not always serve as a sufficient reminder to trigger updating. In our study, we focused on mood state, but it is also possible that mood alone may not be sufficient. Rather, it is the state+context cue compound that is likely driving the effects found here.

One puzzling aspect of this study is that there were no significant state effects on accurate recall. It is unclear why we found state effects on one measure and not the other. It would be useful to know whether state effects are the same in free recall and source recall (i.e., list discrimination), but we were unable to find such studies in the English literature. Nonetheless, the null effect raises questions about the strength of the manipulation, with the possibility that some measures are more sensitive to it than are others. Perhaps a stronger manipulation, such as a drug-induced state, would produce an effect that would be evident in both measures.

One difference between the current and previous studies is that we used words whereas studies by Hupbach and colleagues used objects as memoranda. It is possible that objects are better bound to environmental context conditions than are words (e.g., the overshadowing hypothesis, Smith & Vela, 2001; but see Kiley & Parks, 2020 for similar experiments with words). If so, then the updating pattern that we failed to find here when spatial context was constant, but mood was not, could appear with objects. However, such a finding would still imply that it is context effects at play, rather than reconsolidation (e.g., Gisquet-Verrier & Riccio, 2019b).

One limitation of the current study is that support for the integration concept relies in part on null effects, specifically, the lack of a difference between AI scores and zero in the *different* conditions. Although the results favor the integration concept more so than the reconsolidation theory (especially when directly comparing the *same* vs. *different* conditions, ***Figure 4***), replications of these findings would be useful to solidify that interpretation. For instance, it would be useful to examine the effects of mood in the current conditions and in conditions without a spatial reminder. It would also be informative to replicate the current study with two *same* conditions in a single experiment (i.e., all neutral and all negative in one experiment). Finally, it could be the case that List 1 is less likely to intrude on List 2 simply because it has a special status as being the first list encountered (and potentially being the first list ever encountered in an experimental setting). Thus, it would be informative to test three lists rather than two, but to ignore the first list and compare only Lists 2 (presented on Day 1) and 3 (presented on Day 2). If List 1 is special in that it is less likely to intrude on other lists, it could be the case that asymmetrical intrusions would disappear when looking at Lists 2 and 3, calling the memory updating explanation of asymmetrical intrusions into question.

In sum, the current results support the integration concept of memory updating (Gisquet-Verrier & Riccio, 2018, 2019a) in episodic memory. To our knowledge, this is the first test of this concept in human declarative memory. To advance theories of memory updating, further research in humans should investigate the role of time-dependency to test the idea that memory updating is a rapid process, rather than one that requires a rest period to appear (see Gisquet-Verrier & Riccio, 2012 for a review of findings from animals that indicate rapid updating is typical in several paradigms). Though there is some evidence of this in human declarative memory (Bridge & Paller, 2012; Bridge & Voss, 2014), the two theories have not yet been pitted against each other in a single experiment looking at the time assumptions. Additional studies comparing the two theories may be especially important for translational purposes given the interest in using reconsolidation blockades to treat emotional disorders and drug-related memories. There are somewhat mixed results in the human literature on the success of reconsolidation therapies to treat maladaptive memories (Beckers & Kindt, 2017). There is also a publication bias in the retrograde amnesia literature in rodents in contextual fear memory paradigms, indicating that sizes of such weakening effects are not as large as the literature has suggested (Schroyens et al., 2020). Schroyens et al. suggest that the mixed results in the human literature may therefore reflect the fact that memory-weakening effects are not as robust as once thought. If reconsolidation is a not a unique neurobiological process at all, and weakening fear memories is achieved through state manipulations, as argued by the memory integration concept, pursuing reconsolidation as a means of treating maladaptive memories may not be as fruitful as it was once thought to be.

## Data Accessibility Statement

The data collected in these experiments are available by emailing *colleen.parks@unlv.edu* and on the Open Science Framework. The study was not preregistered.
